# Supplementary material for: Automated CT Analysis of Major Forms of Interstitial Lung Disease
Source: J Clin Med. 2020 Nov 23;9(11):3776. doi: 10.3390/jcm9113776 (PMC7700631; doi:10.3390/jcm9113776)
Supplement: Supplementary file 1 [file jcm-09-03776-s001.pdf]

Supplementary

Table 1. ANOVA analysis of global volumes of specific CT findings across multidisciplinary diagnoses.

CT feature	ILD type	Average (mL)	ANOVA *p*-value
Reticulation	IPF	165.6	0.464
	IPAF	169.2	
	CTD	140.8	
	HP	138.5	
Low attenuation	IPF	49.3	0.657
	IPAF	55.5	
	CTD	64.5	
	HP	26.7	
Ground-glass opacity	IPF	784	0.247
	IPAF	846.5	
	CTD	631.8	
	HP	752.4	
Honeycombing	IPF	10.4	0.819
	IPAF	16.7	
	CTD	8.2	
	HP	12.8	

IPF = Idiopathic pulmonary fibrosis; IPAF = Interstitial pneumonia with autoimmune features; CTD = Connective tissue disease; HP = Hypersensitivity pneumonitis.

Table 2. (**a**). ANOVA analysis of left lung central volumes of specific CT findings across multidisciplinary diagnoses. (**b**). ANOVA analysis of right lung central volumes of specific CT findings across multidisciplinary diagnoses.

(**a**)			
**CT feature**	**ILD type**	**Average (mL)**	**ANOVA *p*-value**
Reticulation	IPF	13.3	0.666
	IPAF	18	
	CTD	13.6	
	HP	16.2	
Low attenuation	IPF	10	0.22
	IPAF	19.6	
	CTD	28.2	
	HP	1.1	
Ground-glass opacity	IPF	112.4	0.125
	IPAF	146.5	
	CTD	90.6	
	HP	130.7	
Honeycombing	IPF	1.94	0.675
	IPAF	3.28	
	CTD	0.971	
	HP	3.35	
(b)			
**CT feature**	**ILD type**	**Average (mL)**	**ANOVA *p*-value**
Reticulation	IPF	20.5	0.891
	IPAF	22.5	
	CTD	24.5	
	HP	23.1	
Low attenuation	IPF	18.6	0.525

	IPAF	14.6	
	CTD	15.1	
	HP	4.96	
Ground-glass opacity	IPF	124.3	0.548
	IPAF	155.8	
	CTD	128.1	
	HP	149.9	
Honeycombing	IPF	2.71	0.729
	IPAF	4.46	
	CTD	1.84	
	HP	3.06	

IPF = Idiopathic pulmonary fibrosis; IPAF = Interstitial pneumonia with autoimmune features; CTD = Connective tissue disease; HP = Hypersensitivity pneumonitis.

Table 3. (**a**). ANOVA analysis of left lung peripheral volumes of specific CT findings across multidisciplinary diagnoses. (**b**). ANOVA analysis of right lung peripheral volumes of specific CT findings across multidisciplinary diagnoses.

(**a**)

CT feature	ILD type	Average (mL)	ANOVA *p*-value
Reticulation	IPF	53.1	0.449
	IPAF	55.2	
	CTD	45.1	
	HP	43.8	
Low attenuation	IPF	10.6	0.915
	IPAF	9.27	
	CTD	12.7	
	HP	8.04	
Ground-glass opacity	IPF	264.4	**0.022**
	IPAF	264.8	
	CTD	180.8	
	HP	221.4	
Honeycombing	IPF	2.88	0.932
	IPAF	3.87	
	CTD	2.3	
	HP	3.42	

(**b**)

CT feature	ILD type	Average (mL)	ANOVA *p*-value
Reticulation	IPF	78.7	**0.026**
	IPAF	73.5	
	CTD	57.7	
	HP	55.4	
Low attenuation	IPF	10.1	0.956
	IPAF	12.1	
	CTD	8.53	
	HP	12.6	
Ground-glass opacity	IPF	282.8	0.33
	IPAF	279.4	
	CTD	232.2	
	HP	250.4	
Honeycombing	IPF	2.91	0.782
	IPAF	5.07	
	CTD	3.04	
	HP	3.01	

IPF = Idiopathic pulmonary fibrosis; IPAF = Interstitial pneumonia with autoimmune features; CTD = Connective tissue disease; HP = Hypersensitivity pneumonitis. The bolded values represent the significant results.

Table 4. (**a**). ANOVA analysis of left lung lower zone volumes of specific CT findings across multidisciplinary diagnoses. (**b**). ANOVA analysis of right lung lower zone volumes of specific CT findings across multidisciplinary diagnoses.

(**a**)

CT feature	ILD type	Average (mL)	ANOVA *p*-value
Reticulation	IPF	28.2	0.113
	IPAF	32.9	
	CTD	28.5	
	HP	19.8	
Low attenuation	IPF	3.09	0.786
	IPAF	4.7	
	CTD	1.44	
	HP	2.3	
Ground-glass opacity	IPF	154.8	0.042
	IPAF	162.2	
	CTD	125.2	
	HP	119.8	
Honeycombing	IPF	1.45	0.787
	IPAF	3.05	
	CTD	2.5	
	HP	1.75	

(b)

CT feature	ILD type	Average (mL)	ANOVA *p*-value
Reticulation	IPF	39.5	0.055
	IPAF	41.2	
	CTD	36.9	
	HP	27.1	
Low attenuation	IPF	0.047	0.5
	IPAF	1.58	
	CTD	0.145	
	HP	2.49	
Ground-glass opacity	IPF	159.6	0.312
	IPAF	154.8	
	CTD	157.2	
	HP	128.9	
Honeycombing	IPF	1.59	0.626
	IPAF	2.53	
	CTD	2.21	
	HP	1.19	

IPF = Idiopathic pulmonary fibrosis; IPAF = Interstitial pneumonia with autoimmune features; CTD = Connective tissue disease; HP = Hypersensitivity pneumonitis.

Table 5. (**a**). ANOVA analysis of left lung upper zone volumes of specific CT findings across multidisciplinary diagnoses. (**b**). ANOVA analysis of right lung upper zone volumes of specific CT findings across multidisciplinary diagnoses.

(**a**)

CT feature	ILD type	Average (mL)	ANOVA *p*-value
Reticulation	IPF	15.8	0.462
	IPAF	1.7	
	CTD	13.2	

	HP	19.7	
Low attenuation	IPF	15.3	0.168
	IPAF	11.7	
	CTD	28.8	
	HP	3.17	
Ground-glass opacity	IPF	74.1	0.111
	IPAF	88.5	
	CTD	50.2	
	HP	94.5	
Honeycombing	IPF	1.98	0.618
	IPAF	2.11	
	CTD	0.238	
	HP	3.7	

(b)

CT feature	ILD type	Average (mL)	ANOVA *p*-value
Reticulation	IPF	23	0.776
	IPAF	23.1	
	CTD	18.6	
	HP	22.7	
Low attenuation	IPF	18	0.234
	IPAF	8.05	
	CTD	9.86	
	HP	3.36	
Ground-glass opacity	IPF	92.1	0.177
	IPAF	105.5	
	CTD	72.4	
	HP	116.2	
Honeycombing	IPF	2.57	0.797
	IPAF	3.98	
	CTD	1.06	
	HP	3.15	

IPF = Idiopathic pulmonary fibrosis; IPAF = Interstitial pneumonia with autoimmune features; CTD = Connective tissue disease; HP = Hypersensitivity pneumonitis.

Table 6. Patient mortality data compared to normal tissue volume.

Mortality status	Average (mL)	*p*-value
Alive (n = 177)	1667.5	0.323
Deceased (n = 48)	1560.7	
